# Synergistic adsorption of berberine onto ball-milled biochar-montmorillonite composites derived from traditional Chinese medicine residue: performance, mechanism, and regeneration

**DOI:** 10.3389/fchem.2026.1845578

**Published:** 2026-06-04

**Authors:** Wanzheng Ma, Wenqiang Wang, Lanqing Li, Hao Dai, Cong Tu, Jie Zhang, Yue Xie, Zhifan Zhang, Yan Liu

**Affiliations:** 1 College of Resource and Environment, Anhui Science and Technology University, Bengbu, China; 2 College of Chemical Engineering and Resource Utilization, Northeast Forestry University, Haerbin, China; 3 Department of Plant Pathology, North Carolina State University, Raleigh, NC, United States; 4 College of Animal Science, Anhui Science and Technology University, Bengbu, China; 5 Institute of Subtropical Agricultural Ecology, Chinese Academy of Sciences, Changsha, China

**Keywords:** berberine, biochar, co-ball milling, montmorillonite, traditional Chinese medicine residues

## Abstract

The accumulation of root-exuded berberine (BER) in soil is a key contributor to continuous cropping obstacles, threatening sustainable agriculture. In this study, a novel biochar–montmorillonite composite was synthesized via co-ball milling using traditional Chinese medicine residue derived biochar and montmorillonite (Mt) for berberine removal. The composite exhibited superior adsorption performance with a maximum capacity of 231.60 mg·g^-1^. Characterization by Brunauer–Emmett–Teller (BET) surface area analysis, scanning electron microscopy (SEM), X-ray diffraction (XRD), Fourier-transform infrared spectroscopy (FTIR), X-ray photoelectron spectroscopy (XPS), and Raman spectroscopy revealed that co-ball milling created an intimate hybrid structure with enhanced surface area and abundant functional groups. Adsorption kinetics followed a pseudo-second-order model governed by intraparticle diffusion, while isotherms were well-described by Langmuir, Freundlich, and Temkin models. Thermodynamic analysis indicated spontaneous and endothermic adsorption. The optimal pH range for berberine (BER) adsorption onto the BMC-Mt10% composite was 5–7. Relatively low concentrations of dissolved HA (≤10 mg) can enhance the adsorption capacity for BER, and the composite maintained 76.99% of its initial adsorption capacity after five consecutive regeneration cycles. Mechanistic studies elucidated that the adsorption involved multifaceted interactions, including pore filling, hydrogen bonding, π–π stacking, cation exchange, and electrostatic attraction. These findings demonstrate that biochar–montmorillonite composites are promising materials for mitigating berberine-induced continuous cropping obstacles in agricultural soils.

## 1 Introduction

Traditional Chinese medicine has demonstrated remarkable clinical efficacy in the treatment of diseases worldwide (Cao et al., 2020; Wang J. et al., 2019). Driven by national strategic plans such as the “Strategic Planning Outline for the Development of Traditional Chinese Medicine (2016–2030)”, the planting area of medicinal materials in China has exceeded 2.4 million hectares (Wu et al., 2022). With the rapid development of the traditional Chinese medicine industry, the annual output of discarded Chinese medicine residues exceeds 60 million tons (Lu and Li, 2021). Currently, these residues are primarily disposed of through landfilling and incineration (Hou et al., 2022). This not only leads to a decline in soil productivity but also poses a serious threat to surface water safety due to heavy metals and complex chemical components in the leachate, while exacerbating air pollution (Wu et al., 2025). From the perspective of sustainable development and a low-carbon economy, converting Chinese medicine residues into biochar through pyrolysis offers a promising strategy for resource utilization. This approach not only solves the problem of waste disposal but also transforms the residues into functional materials for environmental remediation.

Concurrently, intensive agricultural practices, while achieving high yields, have exacerbated challenges associated with continuous cropping (Fang et al., 2025; Wang J. et al., 2022). Among these, the accumulation of autotoxic allelochemicals in soil is recognized as a key mechanism underlying continuous cropping disorders, adversely affecting seed germination, seedling growth, and overall crop productivity (Lin et al., 2023; Wang F. et al., 2022). Alkaloids represent a prominent class of such allelochemicals, being particularly notorious for severely inhibiting seed germination and seedling growth (Zhao X. et al., 2024). Berberine (BER), a prominent alkaloid, is widely recognized as a pivotal element exacerbating issues associated with continuous cultivation (Fang et al., 2025). Zhang et al. has shown that 7 days of BER exposure markedly suppresses root growth in Eleusine indica, Vetiveria zizanioides, and Mikania micrantha (Zhang et al., 2011). Current strategies for overcoming continuous cropping obstacles primarily involve intercropping and crop rotation (Feng et al., 2021; Zhao X. et al., 2024). While these approaches can improve soil conditions and reduce allelochemical accumulation, their complex management requirements and potential competition between crops may lead to decreased yields (Zhao et al., 2025). Currently, the main techniques for removing BH include biodegradation, electrochemical methods, Fenton oxidation, and adsorption (Li et al., 2014; Moore et al., 2022). Biodegradation exhibits relatively low removal efficiency due to anodic passivation. Although Fenton oxidation can effectively eliminate BH, the process generates toxic intermediates with potential biological hazards. In contrast, adsorption not only overcomes these limitations but also offers advantages such as operational simplicity, low cost, and broad applicability.

Biochar (BC) is widely recognized as a cost-effective and environmentally friendly adsorbent, making it a popular choice for the treatment of both wastewater and soil (Cho et al., 2023; El-Naggar et al., 2019). It reduces the migration and transformation of pollutants in the environment through surface adsorption and distribution (Fseha et al., 2021). Therefore, the use of biochar to adsorb autotoxic substances has a high potential for regulating continuous cropping obstacles. Given the inherently low adsorption capacity of raw biochar, extensive research has been dedicated to improving its performance via various modification techniques and composite formations (Dai et al., 2025a). Biochar-based composites, with their high surface area, good conductivity, and excellent chemical stability, are increasingly recognized for their potential in energy storage and environmental applications (Z et al., 2022; Dai et al., 2025b). It significantly enhances the adsorption efficiency and have attracted considerable attention from researchers due to their exceptional surface active sites and abundant functional groups (Jing et al., 2022; Li et al., 2023). Montmorillonite (Mt) stands out as an economical clay mineral boasting an extensive surface area, a unique layered structure, and impressive adsorption capabilities (Zhu et al., 2019). Notably, Mt has an exchangeable cation interlayer space and interacts with the surface through various oxygen-based functional groups, van der Waals forces, and ion exchange. Therefore, Mt is commonly employed to eliminate organic pollutants from both water and soil environments (Sathasivam et al., 2025). However, its strong hydrophilicity, viscosity, and colloidal characteristics hinder separation and recovery, thereby restricting its adsorption efficiency for pollutants (Benhouria et al., 2015). The porous nature and high retention capacity of BC make it a suitable material for creating a synergistic composite with Mt particles (Chen et al., 2017).

The synthesis methods of biochar composites mainly include: co-precipitation method, impregnation method and ball milling method (Yang et al., 2023). Among these, ball milling is widely recognized as a simple, efficient, and eco-friendly method for enhancing biochar properties, as demonstrated by its ability to improve adsorption capacity, increase surface area, and enrich surface functional groups (Ali et al., 2025). Recent studies have shown that the ball milling process significantly enhances the integration of biochar with macroscopic and nanoscale materials, leading to efficient synergistic effects between the components, thereby significantly optimizing the structure and performance of biochar (Zeng et al., 2024). Liu et al. prepared biochar–vermiculite/zeolite magnetic composite materials via ball milling, which exhibited excellent adsorption performance for Pb^2+^ and PNP removal from water (Liu et al., 2025). Song et al. synthesized zeolite–biochar composite adsorbents through mechanical ball milling, significantly enhancing their adsorption capacity for phosphate (Song et al., 2025). To date, systematic investigations remain scarce on ball-milled biochar–montmorillonite composites derived from traditional Chinese medicine residues, particularly regarding their application in alkaloid adsorption and the underlying synergistic mechanisms.

Therefore, we hypothesize that co-ball milling of biochar and montmorillonite will create a synergistic composite with enhanced adsorption sites and stability, producing superior berberine removal performance compared to a simple physical mixture. The objectives are to: (1) prepare and characterize a series of ball-milled biochar-montmorillonite composites with different Mt loadings; (2) evaluate the adsorption capacity of the optimal composite for BER through batch adsorption experiments, elucidating the effects of various factors including contact time, initial concentration, temperature, pH, and humic acid on the adsorption process; and (3) comprehensively investigate the underlying adsorption mechanisms using advanced spectroscopic techniques including Raman spectroscopy and X-ray photoelectron spectroscopy (XPS) before and after BER adsorption. This research provides novel insights into the design and application of ball-milled clay-biochar composites for alleviating continuous cropping obstacles and offers a sustainable valorization pathway for traditional Chinese medicine residue valorization.

## 2 Materials and methods

### 2.1 Samples and reagents

Traditional Chinese medicine residue biomass was collected from Fengyang Traditional Chinese Medicine Hospital, washed thoroughly with deionized water, and subsequently oven-dried at 60 °C. The dried biomass was ground into a homogeneous powder and sieved for subsequent characterization and biochar preparation. Chromatographic grade solvents, including methanol, acetonitrile, and ethanol, were purchased from Sigma-Aldrich. BER standard (purity > 95%) was obtained from Shanghai Yuanye Bio-Technology Co., Ltd. Mt clay was supplied by Donglong Chemical in Guangxi province. All remaining chemicals were analytical-grade reagents, and ultrapure water served as the solvent throughout the experimental procedures.

### 2.2 Materials preparation

A measured amount of powdered traditional Chinese medicine residue was placed into a crucible and heated in a muffle furnace. Pyrolysis was conducted in air, with the temperature ramped from room temperature to 500 °C at a rate of 10 °C·min^-1^ and maintained for 2 h, followed by natural cooling to room temperature. Once the process concluded, the sample cooled down on its own to ambient temperature before being weighed. From there, the biochar yield was determined, and the final product was labeled simply as BC. To fabricate the montmorillonite-biochar composite material, Mt was added to BC in mass ratios of 10%, 30%, and 50%, as per the findings from the study on the effect of montmorillonite-biochar composite amendment on thallium bioavailability in contaminated agricultural soils. Each mixture was weighed, placed into an agate jar (with a mass ratio of grinding beads to mixture of 100:1), and ground at 300 rpm for 6 h, with the direction of rotation changed every 3 h. The composite materials obtained after co-ball milling were named based on the proportion (BMC-Mt10%, BMC-Mt50% and BMC-Mt50%). Other control materials, BC and Mt ball-milled separately, were designated as BMC and BMt, respectively.

### 2.3 Materials characterization

The elemental composition of the selected adsorbents, including carbon (C), hydrogen (H), and nitrogen (N), was meticulously analyzed using an elemental analyzer (Elementar Vario EL, Elementar, Germany). The surface morphology of these materials was observed using scanning electron microscopy (SEM; EVO-18, Carl Zeiss, Germany), while energy-dispersive X-ray spectroscopy (EDS) was employed to determine their elemental composition. The specific surface area and pore volume of the materials were measured using the Brunauer-Emmett-Teller (BET) method with nitrogen adsorption (ASAP2460, Micromeritics, USA). The crystallinity of the selected adsorbents was determined by X-ray diffraction (XRD; XD-3, Beijing Persee, China), with a scan range (2θ) of 10°–90° and a scan rate of 2°·min^-1^. Fourier transform infrared spectroscopy (FTIR; FTIR-850, Tianjin Gangdong, China) was used to record the functional group distribution, with a range of 4,000–500 cm^-1^. Thermal stability was evaluated via thermogravimetric analysis (TGA) using a Netzsch STA-449 F5 thermal analyzer (Netzsch, Germany). The samples were heated from 35 °C to 900 °C at a constant heating rate of 20 °C·min^-1^. Raman spectroscopy (XploRA-PLUS, HORIBA, Japan) was employed to characterize the functional groups and the degree of graphitization of the adsorbents within a scanning range of 500–2,000 cm^-1^. Furthermore, X-ray photoelectron spectroscopy (XPS) was utilized to analyze the surface elemental composition and chemical states of the adsorbents both before and after the adsorption process.

### 2.4 Adsorption experiments

#### 2.4.1 Method of high-performance liquid chromatography

High-performance liquid chromatography (HPLC) was used to analyze the chromatographic peaks in the solution, and the BER solution concentrations were calculated using the standard curve. Chromatographic conditions: Shim-pack VP-ODS column (250 mm × 4.6 mm, 5 μm); column temperature: 30 °C; detection wavelength: 210 nm; flow rate: 1.0 mL/min; injection volume: 10 μL. Mobile phase A: acetonitrile, mobile phase B: 0.1% phosphoric acid aqueous solution. Isocratic elution was used: 0–5 min, 40% A, 60% B. All solutions were filtered through a 0.22 μm filter membrane before injection. The methodological validation of the HPLC analysis are provided in Supplementary Table S1.

#### 2.4.2 Adsorption performance test

To determine the optimal mass ratio of the BMC-Mt composite, 0.010 g of samples with different proportions were weighed into conical flasks, followed by the addition of 40 mL of BER solution at a concentration of 100 mg·L-1. The flasks were placed in a constant-temperature shaker incubator and shielded from light. The mixtures were oscillated at (25 ± 1) °C and 160 r/min for 24 h, after which samples were taken to determine the residual BER concentration. Each experiment was performed in triplicate.

The adsorption capacity at equilibrium (Q_e_, mg·g^-1^) was calculated using the following mass balance (Equation 1):

$$Q_{e}=\frac{\left(c_{0}-c_{t}\right)V}{m}$$ (1)

where C_0_ and C_e_ (mg·L^-1^) are the initial and equilibrium concentrations of BER, respectively; V (L) is the volume of the BER solution; and m (g) is the mass of the adsorbent used.

#### 2.4.3 Adsorption kinetics experiment

The adsorption kinetics of BER onto the prepared biochar samples were investigated to elucidate the adsorption rate and potential rate-controlling mechanisms. For each kinetic experiment, 0.010 g of adsorbent was weighed into a 100 mL conical flask, followed by the addition of 40 mL of BER solution with an initial concentration of 100 mg·L^-1^. The flasks were then placed in a constant-temperature shaker incubator and agitated at 160 rpm and 25 °C ± 1 °C. At predetermined time intervals (ranging from 0 to 360 min), 1.0 mL aliquots of the suspension were collected, immediately filtered through a 0.22 μm membrane filter, and the BER concentration was determined using HPLC. All experiments were performed in triplicate. The kinetic data were analyzed using three commonly employed models to describe the adsorption process and identify the rate-limiting steps.

The pseudo-first-order kinetic model (PF-order) is expressed as Equation 2:

$$Q_{t}=Q_{e}\left(1-e^{-k_{1}t}\right)$$ (2)

The pseudo-second-order kinetic model (PS-ord) is expressed as Equation 3:

$$Q_{t}=\frac{k_{2}Q_{e}^{2}t}{1+k_{2}Q_{e}t}$$ (3)

The intraparticle diffusion model (IPD) is expressed as Equation 4:

$$Q_{t}=k_{3}t^{0.5}+b$$ (4)

where Qe and Qt represent the adsorption amount of BER at equilibrium and the adsorption amount of BER at time t (mg·g^−1^); k_1_ (min^−1^), k_2_ [g·(mg·min)^−1^] and k_3_ [mg·g^−1^ min^−0.5^] were the rate constants of PF-order, PS-ord and IPD, respectively. b is a constant of the internal diffusion model equation.

#### 2.4.4 Adsorption isotherm experiment

Adsorption isotherm experiments were conducted to evaluate the equilibrium adsorption behavior of BER onto the prepared biochar samples and to determine the maximum adsorption capacity. For each experiment, 0.010 g of adsorbent was weighed into a series of 100 mL conical flasks, followed by the addition of 40 mL of BER solutions with initial concentrations ranging from 40 to 160 mg·L^-1^. The flasks were then placed in a constant-temperature shaker incubator and agitated at 160 rpm for 24 h to ensure complete equilibration. The experiments were performed at three different temperatures (25 °C, 35 °C, and 45 °C) to investigate the temperature dependence of adsorption. After reaching equilibrium, samples were collected and determined using HPLC. All experiments were conducted in triplicate. The equilibrium adsorption data were fitted using three classical isotherm models to elucidate the adsorption characteristics and surface properties of the adsorbents.

The Langmuir isotherm model is expressed as Equation 5:

$$Q_{e}=\frac{q_{m}K_{L}c_{e}}{1+K_{L}C_{e}}$$ (5)

The Freundlich model is expressed as Equation 6:

$$Q_{e}=K_{f}C_{e}^{\frac{1}{n}}$$ (6)

The Temkin model is expressed as Equation 7:

$$Q_{e}=\frac{RT}{b}\ln K_{T}+\frac{RT}{b}\ln c_{e}$$ (7)

where Ce is the concentration at adsorption equilibrium (mg·L^−1^), and Q_e_ is the adsorption capacity at equilibrium (mg·g^−1^). K_L_ is the adsorption constant of Langmuir model (L·mg^−1^). K_f_ is the Freundlich model adsorption constant (mg^1-1/n^·L^1/n^·g^-1^), n is the heterogeneity constant; K_T_ is the equilibrium binding constant (L·g^-1^) of Temkin model, b is the constant of Temkin model, R is the constant of ideal gas, and T is the thermodynamic temperature(K).

#### 2.4.5 Adsorption thermodynamics

To gain insights into the thermodynamic nature of BER adsorption onto the prepared materials, thermodynamic parameters including Gibbs free energy change (Δ*G*°), enthalpy change (Δ*H*°), and entropy change (Δ*S*°) were calculated from the temperature-dependent adsorption data. The thermodynamic parameters were determined using the following Equations 8, 9 based on the Langmuir equilibrium constant

$$\ln K_{L}=-\frac{\Delta H^{\theta }}{RT}+\frac{\Delta S^{\theta }}{R}$$ (8)

$$\Delta G^{\theta }=\Delta H^{\theta }-T\Delta S^{\theta }$$ (9)

where the adsorption thermodynamic parameters Δ*H* ^θ^ (kJ·mol^-1^) are enthalpy change, Δ*S* ^θ^(J·K·mol^-1^) is entropy change, and Δ*G* ^θ^ (kJ·mol^-1^) is Gibbs free energy change. R is the ideal gas constant, T is the thermodynamic temperature (K), and K_L_ is the Langmuir equation constant (L·mol^-1^).

#### 2.4.6 Regeneration performance test

The biochar saturated with BER was placed in 0.5 mol·L^-1^ NaOH solution and subjected to ultrasonic-assisted desorption for 12 h. After desorption, the biochar was rinsed with deionized water until neutral pH, dried, and reused in the subsequent adsorption experiment. Five consecutive adsorption–desorption cycles were conducted, and the adsorption capacity was measured after each cycle.

#### 2.4.7 Effect of initial pH and humic acid on adsorption

A total of 0.010 g of biochar was placed in a conical flask, and 40 mL of BER solution with an initial concentration of 100 mg·L^-1^ was added. The initial pH of the mixture was adjusted to values ranging from 3.0 to 11.0 (specifically 3.0, 5.0, 7.0, 9.0, and 11.0) using 1.0 mol·L^-1^ HCl or NaOH solutions. The flask was then incubated in a light-shielded constant-temperature shaker at (25 ± 1) °C and 160 r/min for 24 h before sampling and analysis.

A total of 0.010 g of biochar was weighed into a conical flask, and 40 mL of a mixed solution containing 100 mg/L BER and different concentrations of humic acid was added. The flask was kept in the dark and oscillated in a constant-temperature shaker at (25 ± 1) °C and 160 r/min for 24 h. After adsorption, samples were taken to determine the residual BER concentration.

### 2.5 Data analysis

The adsorption capacity (Q_e_, mg·g^-1^) was calculated based on the concentration difference between the initial solution (C_0_, mg·L^-1^) and the equilibrium solution (C_e_, mg·L^-1^). The adsorption kinetics were modeled using the pseudo-first-order, pseudo-second-order, Elovich, and intraparticle diffusion equations. The adsorption isotherms were analyzed with the Langmuir, Freundlich, and Temkin models. The detailed characterization of the support materials and the experimental data in this study were acquired via High-Performance Liquid Chromatography (HPLC) with LabSolutions software, and the data were subsequently exported in text file format (*.txt). Subsequently, the raw data were visualized utilizing Origin2022 software.

## 3 Results and discussion

### 3.1 Screening and synergistic adsorption performance of composites

**FIGURE 1:**
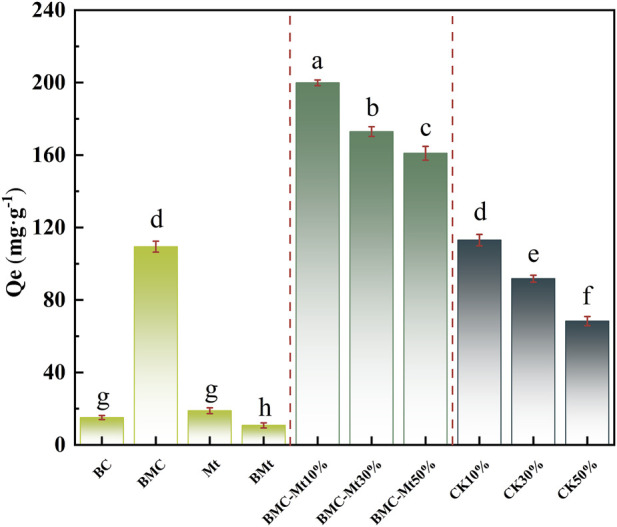
Comparative analysis of adsorption performance among various adsorbents.

The adsorption capacities of the pristine and modified materials for BER are compared in Figure 1. Among all synthesized materials, BMC-Mt10% exhibited the highest adsorption capacity. For comparison, the BC and Mt displayed significantly lower capacities of 15.08 and 18.89 mg·g^-1^, respectively. Ball milling markedly altered their individual performances: the adsorption capacity of BMC increased by a factor of 7.3, while that of ball-milled Mt decreased by a factor of 1.8. When the materials were composited via co-ball milling, the resulting adsorption performance became highly dependent on the Mt content. Among the composites, BMC-Mt10% exhibited the highest adsorption capacity, reaching 199.8 mg·g^-1^. This represents a 13.26-fold and 10.58-fold increase compared to pristine BC and Mt, respectively. Zhao et al. reported that a magnesium-modified biochar exhibited a maximum BER uptake of 113.89 mg·g^-1^, whereas BMC-Mt10% in this study achieved 199.8 mg·g^-1^ (Zhao Y. et al., 2024). This enhancement highlights the synergistic effect induced by mechanochemical co-ball milling of traditional Chinese medicine residue and Mt. However, the capacity decreased significantly to 84.1 and 52.4 mg·g^-1^ for BMC-Mt20% and BMC-Mt30%, respectively, indicating that indicating that an optimal loading exists for maximizing the synergistic effect. This non-linear trend indicates a significant synergistic effect rather than a simple additive one. This conclusion is supported by comparison with a physical mixture of separately ball-milled BC and Mt (10 wt.%), which exhibited a capacity of only 15.2 mg·g^-1^. BMC-Mt10% outperforms BMC + Mt10% by more than an order of magnitude, demonstrating that the enhancement arises from the novel interface and structure formed during co-ball milling. We propose that an optimal Mt content (10%) enables effective dispersion and integration within the biochar matrix, thereby creating new active sites. Consequently, BMC-Mt10% was selected for subsequent characterization and mechanistic studies, with BMC and BC used as reference materials.

### 3.2 Structural and property characterization of materials

#### 3.2.1 Morphology and pore structure analysis

**FIGURE 2:**
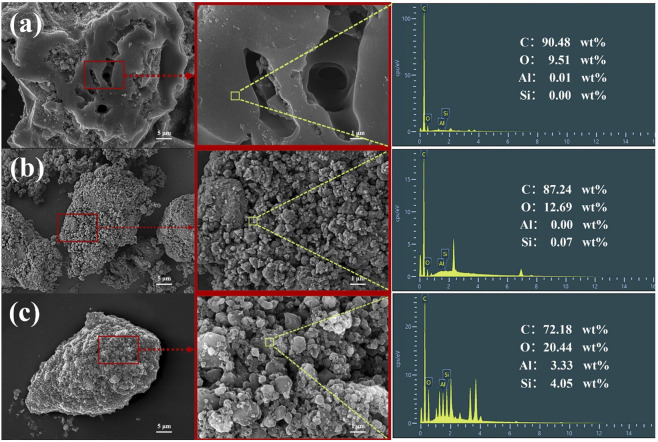
Illustrates the SEM images of **(a)** biochar (BC), **(b)** biochar-montmorillonite composite (BMC), and **(c)** BMC with 10% montmorillonite (BMC-Mt10%). The corresponding EDS analysis on the right side of each SEM image depicts the elemental composition of the respective materials, highlighting the distribution of elements within the composites.

The surface morphology and microstructural evolution of BC, BMC, and the optimal composite BMC-Mt10% were investigated using SEM and EDX (Figure 2). The BC exhibited a relatively smooth surface, retained the original skeletal structure of the biomass feedstock, and displayed a porous network with distinct channels (Figure 2a). This microstructure originates from the decomposition of biopolymers (e.g., cellulose and lignin) and the release of volatile compounds during pyrolysis (Vakili et al., 2023). Ball milling induced significant morphological changes. The surface of BMC became markedly rugged and granular (Figure 2b). This change is attributed to intense mechanical friction and collisions during ball milling, which fracture larger carbon structures into finer, irregular particles (Lyu et al., 2018). The most notable transformation was observed in BMC-Mt10% (Figure 2c). Its surface distinctly displayed a layered and sheet-like structure, markedly different from both BC and BMC. This morphology provides direct evidence that Mt layers were effectively exfoliated and integrated into the biochar matrix during co-ball milling, resulting in a novel composite structure. This intimate interface formed during co-ball milling is critical for the synergistic effect, as it maximizes the contact area between the two components and may generate new adsorption sites. EDX analysis and elemental composition data (Supplementary Table S2) provide evidence for successful composite formation. While BC and BMC consist primarily of C and O, the spectrum of BMC-Mt10% reveals the presence of Si, Al, and Mg (Figure 2c inset). These elements are characteristic of the Mt, further confirming the integration of Mt into the carbonaceous matrix (Dai et al., 2025a). The quantitative data in Supplementary Table S2 show a significant increase in Si and Al content and a corresponding decrease in C content in BMC-Mt10%, attributable to the incorporation of the clay mineral. Furthermore, elemental analysis indicates that ball milling alters the surface chemistry of biochar. The increase in O content from 42.66% in BC to 45.03% in BMC suggests that the mechanical process exposes additional carbon edge sites and promotes the formation or enhancement of oxygen-containing functional groups, which may serve as active adsorption sites (Tang et al., 2021). The changes in atomic ratios (e.g., O/C, (O + N)/C) in BMC-Mt10% indicate modified surface polarity and hydrophilicity compared to its precursors, which may influence its interaction with organic contaminants such as BER (Yang Y. et al., 2018).

**FIGURE 3:**
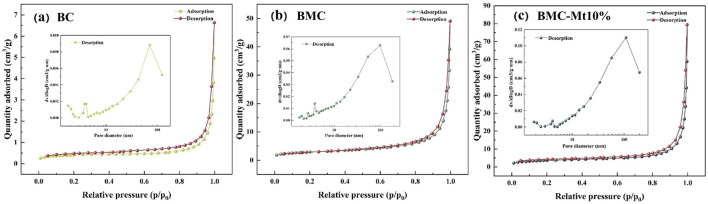
Illustrates the nitrogen adsorption-desorption isotherms and pore volume distribution for **(a)** BC, **(b)** BMC, and **(c)** BMC-Mt10%, indicating the materials' specific surface area and pore structure characteristics.

The pore structure characteristics of BC, BMC, and BMC-Mt10% were analyzed using N_2_ adsorption-desorption, with isotherms and pore size distributions presented in Figure 3 and quantitative data summarized in Table 1. All three materials exhibit Type IV isotherms with H4-type hysteresis loops, which are typical for mesoporous biochar-based materials and indicate the presence of slit-shaped pores (Mariana et al., 2021). The pore size distribution curves indicate that the porosity is predominantly composed of mesopores (2–50 nm) and macropores (>50 nm). Ball milling induced significant changes in the textural properties. BC exhibits a specific surface area (SSA) of 1.39 m^2^·g^-1^, whereas the SSA of BMC increases to 4.73 m^2^·g^-1^. Notably, BMC-Mt10% exhibits an SSA of 12.71 m^2^·g^-1^ and a total pore volume of 0.113 cm^3^·g^-1^, corresponding to 9.1-fold and 10.9-fold increases over BC, respectively. This enhancement in SSA and pore volume corroborates the morphological changes observed by SEM, where co-ball milling creates a finely integrated and rugged layered structure. The pore structure evolution provides critical insight into the adsorption performance. The increased SSA and pore volume of BMC-Mt10% provide a greater number of potential adsorption sites and improved mass transfer pathways for BER molecules. The H4-type hysteresis loop observed for BMC-Mt10% differs from the H3-type commonly reported for Mt (Yaghmaeiyan et al., 2022). This shift suggests that the classic slit-shaped pores between Mt platelets have been modified. This change is attributed to the co-ball milling process, which integrates clay layers into the carbon matrix and likely creates a more complex hybrid pore system composed of biochar-derived pores and clay–carbon interfacial pores. The optimized pore structure of BMC-Mt10%, featuring enhanced surface area and a mix of pore sizes, is a key factor contributing to its superior adsorption capacity, as it improves both the diffusion of BER molecules and their attachment to active sites.

**TABLE 1:** Surface properties of BC, BMC and BMC-Mt10%.

Biochar	S_BET_	S_mic_	S_mes_	V_tot_	V_mic_	D_avg_	D_mes_
BC	1.397	0.554	0.843	0.007	0.000266	20.35	32.56
BMC	10.01	2.277	7.731	0.059	0.001062	23.68	24.07
BMC-Mt10%	12.71	2.963	9.749	0.077	0.112848	24.27	33.17

SBET: specific surface area (m^2^/g) by BET, method; Smic: micropore surface area (m^2^/g); Smes: mesopore surface area (m^2^/g); Vtot: total pore volume (cm^3^/g); Vmic: micropore volume (cm^3^/g); Davg: average pore diameter (nm); Dmes: mesopore diameter (nm).

#### 3.2.2 Surface chemistry and crystalline structure analysis

**FIGURE 4:**
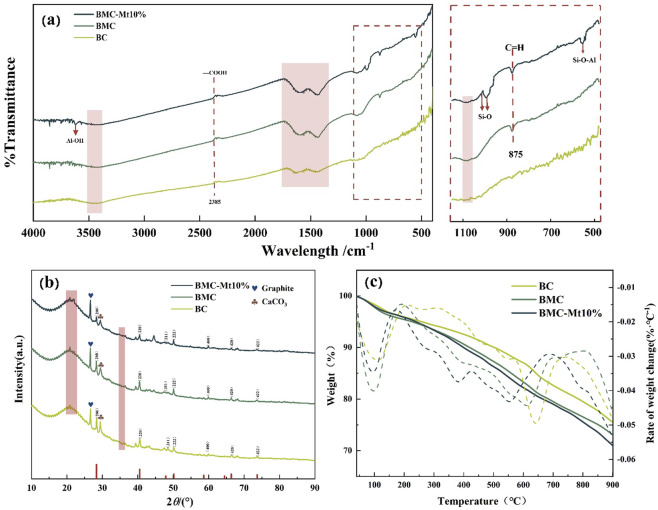
Characterization spectroscopy of selected adsorbents BC, BMC and BMC-Mt10% by FTIR **(a)** and XRD **(b)** and TGA **(c)**.

Figure 4a displays the FT-IR spectra of the three materials, which show similar infrared absorption peaks, suggesting comparable functional group structures. The broad peak in the 3,410–3,510 cm^-1^ range is attributed to the stretching vibration of -OH hydrogen bonds (Chen et al., 2008). The peak at 2,358 cm^-1^ corresponds to the–OH stretching vibration of carboxylic acid dimers, indicating the presence of–COOH groups. In the 1,350–1,750 cm^-1^ range, absorption peaks corresponding to C=O and C=C stretching vibrations in aromatic rings are observed (Chen et al., 2023), and these peaks intensified after ball milling. Around 1,060 cm^-1^, a symmetric C–O–C stretching vibration is observed, characteristic of cellulose-derived structures in biomass after pyrolysis. This peak becomes more prominent after ball milling, as the process exposes surface oxygen-containing functional groups (Lyu et al., 2018). The bending vibration of aromatic C-H bond at 875 cm^-1^ suggests that ball milling enhances the number of aromatic functional groups. In BMC-Mt10%, the incorporation of Mt results in a characteristic Al–OH absorption peak at 3,619 cm^-1^, originating from its octahedral structure. The absorption peaks at 996 and 1,115 cm^-1^ are attributed to the stretching vibrations of Si-O. A Si–O–Al absorption peak at 553 cm^-1^ further indicates that the interaction between Mt and BC is not limited to simple surface attachment. These changes in surface properties contribute to the enhanced removal of organic pollutants from aqueous systems (Xu et al., 2018).

Figure 4b presents the XRD spectra of the three materials. All three materials exhibit a broad amorphous peak within the 2θ range of 10°–30° (Shu et al., 2010). All three materials also exhibit sharp diffraction peaks at 28.3° and 40.4°, indicating a predominantly carbon-based crystalline structure (Liou and Wu, 2009). The biomass derived from traditional Chinese medicine contains significant amounts of potassium, which forms KCl or other potassium-containing minerals during high-temperature pyrolysis. This corresponds to the characteristic peaks of the KCl crystal structure in the figure, with diffraction peaks at 200°, 220°, 222°, and 420° (Yang X. et al., 2018). In contrast, the peaks of the composite materials are reduced in intensity. This reduction is attributed to the lower biomass content in the composite materials. After co-ball milling, the crystal structure is further disrupted, as evidenced by changes in the CaCO_3_ peak at 2θ = 29.5° in BMC-Mt10%. The diffraction peaks of Mt typically appear near 2θ = 19.96° and 32.02° (Ejder-Korucu et al., 2016). However, in the XRD spectrum of BMC-Mt10%, these peaks are less pronounced, with no significant shift in diffraction angles. This may result from alterations in the 2:1 layered structure of Mt during ball milling, rather than simple surface attachment with retention of its original structure. This structural disruption likely exposes more internal surface area and active sites, contributing to the enhanced adsorption capacity.

#### 3.2.3 Thermal stability analysis

The thermal stability and decomposition behavior of BC, BMC, and BMC-Mt10% were evaluated by TGA and DTG under N_2_ atmosphere (Figure 4c). The results show that none of the materials exhibit significant weight loss when heated to 900 °C, indicating high thermal stability under inert conditions. The total weight losses of BC, BMC, and BMC-Mt10% were 24.84%, 27.25%, and 29.34%, respectively. This stability is expected, as the biochar component has already undergone devolatilization and carbonization during its initial pyrolysis at 500 °C under oxygen-limited conditions (Kumar et al., 2020), where the decomposition of cellulose, hemicellulose, and lignin largely occurred. Despite this stability, the slightly higher weight loss of BMC (27.25%) compared to BC (24.84%) is attributable to the ball milling process. Ball milling disrupted the stable carbon framework, exposing oxygen-containing functional groups on the surface. Upon heating, these newly exposed and less stable groups undergo further dehydrogenation and deoxygenation, resulting in incremental mass loss (Nolte and Shanks, 2017). Notably, BMC-Mt10% exhibits the highest total weight loss (29.34%) among the three materials. This additional mass loss is attributed to the incorporation of Mt. The DTG curve of BMC-Mt10% shows a distinct peak between 50 °C and 180 °C, corresponding to the evaporation of interlayer-bound and adsorbed water from Mt, which accounts for the increased weight loss. The presence of this peak confirms that the hydration propertie of Mt is preserved in the composite, even after the co-ball milling. Furthermore, the disruption of the clay structure during ball milling (Figure 4b) may expose additional hydroxylated edges, further contributing to the overall mass loss profile.

### 3.3 Adsorption behavior and mechanism investigation

#### 3.3.1 Adsorption kinetics

**FIGURE 5:**
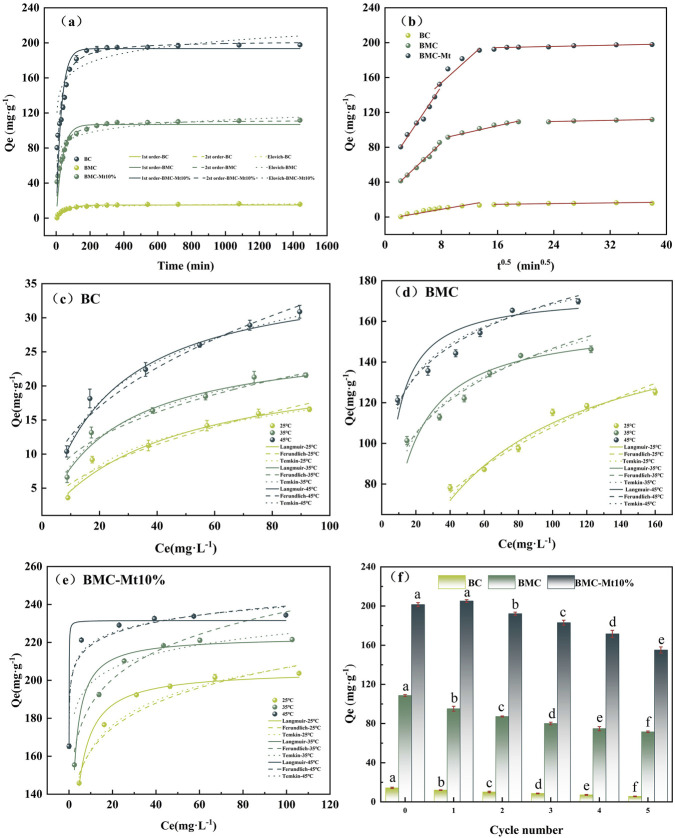
Adsorption performance of biochar-montmorillonite composites: **(a)** kinetic behavior, **(b)** intra-particle diffusion mechanisms, **(c–e)** equilibrium adsorption isotherms, and **(f)** regeneration performance.

The adsorption kinetics of BER onto BC, BMC, and BMC-Mt10% were investigated to elucidate the adsorption rate and potential rate-controlling steps, with the results presented in Figure 5a and Table 2. As shown in Figure 5a, the adsorption of BER onto both BMC and BMC-Mt10% occurs rapidly within the initial 30 min, with over 50% of the equilibrium capacity achieved during this period. This rapid initial uptake indicates a strong driving force for adsorption, attributed to abundant pore structures (Figure 3; Table 1) and exposed surface functional groups (Figure 4a), which provide ample adsorption sites (Petrovic et al., 2022). Following this rapid phase, the adsorption rate gradually decreases as available sites become occupied and surface BER concentration increases, reaching equilibrium at approximately 180 min. The slower later stage is likely influenced by increased electrostatic repulsion between adsorbed and free BER molecules, as well as intraparticle diffusion into deeper pores (Li et al., 2023). The kinetic data were fitted using pseudo-first-order, pseudo-second-order, and Elovich models, with the calculated parameters summarized in Table 2. For BC, the pseudo-first-order model provides the best fit (R^2^ > 0.99), suggesting that physical adsorption or diffusion processes may dominate. This is consistent with the relatively smooth surface and simple pore structure of BC observed in SEM (Figure 2), which limit the number of available adsorption sites and result in lower adsorption capacity. Interestingly, the Elovich model fitting coefficient for BC (R^2^ = 0.886) is higher than those for BMC (R^2^ = 0.777) and BMC-Mt10% (R^2^ = 0.768). Since the Elovich model describes heterogeneous chemisorption on energetically different surfaces (Wu et al., 2009), this higher coefficient for BC may indicate that despite its lower capacity, the adsorption process on BC still involves surface heterogeneity. In contrast, both BMC and BMC-Mt10% are best described by the pseudo-second-order kinetic model, with high correlation coefficients (R^2^ > 0.99). This finding indicates that chemisorption is the rate-limiting step for these modified materials, involving electron sharing or transfer between adsorbent and adsorbate (Ma et al., 2020). The superior fit of the pseudo-second-order model for BMC and BMC-Mt10% is consistent with their more complex surface chemistry, including abundant oxygen-containing functional groups exposed by ball milling (Figure 4a) and, for BMC-Mt10%, additional active sites contributed by integrated clay layers. The adsorption process likely involves multiple mechanisms, including hydrogen bonding, electrostatic interactions, and π-π conjugation, which collectively contribute to the enhanced adsorption capacity and rate of these materials (Sathasivam et al., 2025).

**TABLE 2:** Fitting parameters of intraparticle diffusion model.

Biochar	Pseudo-first-order			Pseudo-second-order			Elovich		
	Q_e_	k_1_	R^2^	Q_e_	k_2_	R^2^	α	β	R^2^
BC	14.99	0.017	0.989	15.72	0.002	0.938	8.028	0.538	0.886
BMC	106.8	0.028	0.814	112.1	5.218	0.938	2746	0.112	0.777
BMC-Mt10%	197.2	0.033	0.836	205.5	3.084	0.930	7970	0.065	0.768

Qe: adsorption capacity at equilibrium (mg/g); k1: pseudo-first-order rate constant (min^-1^); k_2_: pseudo-second-order rate constant (g·mg^-1^·min^-1^); α, β: Elovich constants; R^2^: correlation coefficient.

To further elucidate the diffusion mechanisms, the intraparticle diffusion model was applied, with the results shown in Figure 5b and Supplementary Table S3. For all three materials, the adsorption process can be divided into three stages. The first stage (sharp initial rise) corresponds to external or film diffusion, during which BER molecules migrate through the boundary layer to the external surface of the adsorbent. The second stage represents gradual intraparticle diffusion, during which BER molecules diffuse within the pore structure of the material (Kalak et al., 2022). The third stage is the final equilibrium phase, characterized by slow adsorption as BER diffuses into mesopores and micropores, representing the primary rate-controlling step. Importantly, as shown in Supplementary Table S3, the intercept C of the intraparticle diffusion plots is non-zero for all stages and materials. According to diffusion theory, if intraparticle diffusion were the sole rate-limiting step, the plot should pass through the origin. The non-zero intercepts indicate that intraparticle diffusion is not the sole rate-controlling mechanism (Xing et al., 2023; Yao et al., 2014). This finding corroborates the pseudo-second-order model results, indicating that the adsorption process is complex and involves multiple rate-controlling steps, including film diffusion and surface adsorption. The combined effects of these diffusion pathways and the chemisorption mechanisms discussed above govern the overall adsorption kinetics of BER onto these materials.

#### 3.3.2 Adsorption isotherms

The adsorption isotherms of BER onto BC, BMC, and BMC-Mt10% at 298, 308, and 318 K were analyzed using Langmuir, Freundlich, and Temkin models. The fitting curves are presented in Figures 5c–e, and the corresponding parameters are summarized in Table 3.

**TABLE 3:** Presents the fitting parameters for the isothermal adsorption of BC, BMC, and BMC-Mt10% using BET, Langmuir, and Freundlich models.

Biochar	T/K	Langmuir			Freundlich			Temkin		
		Q_m_	K_L_	R_L_^2^	1/n	K_F_	R_F_^2^	K_T_	b	R_T_^2^
BC	298	24.56	0.023	0.986	0.505	1.774	0.973	0.238	464.5	0.980
	308	27.05	0.042	0.972	0.369	4.144	0.944	0.401	426.2	0.978
	318	37.19	0.045	0.982	0.420	4.817	0.976	0.403	312.9	0.997
BMC	298	170.1	0.018	0.955	0.385	18.32	0.956	0.162	63.15	0.955
	308	160.5	0.087	0.853	0.208	56.28	0.921	2.768	98.62	0.918
	318	173.8	0.195	0.789	0.149	85.20	0.942	24.61	122.6	0.934
BMC-Mt10%	298	205.4	0.508	0.982	0.104	128.0	0.933	577.4	131.4	0.956
	308	223.0	0.877	0.951	0.101	148.0	0.911	389.1	225.6	0.828
	318	231.6	71.09	0.930	0.039	199.5	0.903	314.3	2.112	0.934

Q_m_: maximum adsorption capacity (mg/g); K_L_: Langmuir constant (L/mg); 1/n, K_F_: Freundlich constants; K_T_, b: Temkin constants; R^2^: correlation coefficient.

The adsorption behavior varies significantly among the three materials. For BC, the Langmuir model provides the best fit across all temperatures (R^2^ = 0.972–0.986), slightly outperforming the Freundlich model (R^2^ = 0.944–0.976). This suggests that BER adsorption onto BC predominantly follows a monolayer mechanism on a relatively homogeneous surface (Jung et al., 2015). This observation aligns well with the SEM and kinetic findings for BC, which revealed a relatively smooth surface and simpler pore structure, with the Elovich model suggesting monolayer chemisorption (Figure 5a; Table 2). The maximum monolayer adsorption capacity (Q_m_) of BC increases from 24.56 mg·g^-1^ at 298 K to 37.19 mg·g^-1^ at 318 K, indicating that higher temperatures favor adsorption.

In contrast, BMC shows a better fit to the Freundlich model (R^2^ = 0.921–0.956) than to the Langmuir model (R^2^ = 0.789–0.955). This suggests that ball milling increases surface heterogeneity, leading to multilayer adsorption on energetically diverse sites. The Freundlich heterogeneity factor (1/n) for BMC ranges from 0.149 to 0.385, all below 0.5, indicating favorable adsorption (Cao et al., 2019). The KF value, reflecting adsorption capacity, increases markedly with temperature, indicating that elevated temperatures enhance BER uptake. This transition from Langmuir-type to Freundlich-type behavior after ball milling is attributed to mechanical disruption of the carbon structure, resulting in a more complex and heterogeneous surface with diverse pore geometries and exposed functional groups, as evidenced by SEM and BET analyses (Figures 2, 3; Table 1).

For BMC-Mt10%, the Langmuir model provides excellent fits (R^2^ = 0.930–0.982), while the Freundlich model also shows good correlation (R^2^ = 0.903–0.933). The high Q_m_ values for BMC-Mt10% (205.4–231.6 mg·g^-1^) represent a substantial enhancement compared to BC and BMC, consistent with the screening results in Figure 1. Notably, the KL constant in the Langmuir model for BMC-Mt10% increases dramatically with temperature, particularly at 318 K, indicating strong affinity between the adsorbent and BER at higher temperatures. However, the strong performance of both models suggests that the adsorption mechanism for BMC-Mt10% is more complex than simple monolayer adsorption. This complexity likely stems from the hybrid nature of the composite, where the integrated Mt layers introduce additional adsorption sites and mechanisms, including cation exchange and specific interactions with clay mineral surfaces (Lin et al., 2012). The Freundlich parameters for BMC-Mt10% provide strong evidence for its superior adsorption performance. The KF values are substantially higher than those of BC and BMC, confirming the enhanced adsorption capacity of the composite. Moreover, the 1/n values for BMC-Mt10% are very low, well below 0.5, indicating favorable adsorption and pronounced surface heterogeneity (Cao et al., 2019).

The Temkin model yielded fitting coefficients (R2) close to unity for all materials, with the highest value reaching 0.997 for BC at 318 K. The Temkin model assumes that the heat of adsorption decreases linearly with surface coverage due to increasing adsorbent–adsorbate interactions. The excellent fit of this model, particularly for BC and BMC-Mt10%, indicates that electrostatic interactions or ion exchange processes play an important role in adsorption (Yang Y. et al., 2018). This observation is consistent with the kinetic results (Figure 5a; Table 2), which identified chemisorption as the rate-limiting step for BMC and BMC-Mt10%, and aligns with the anticipated role of oxygen-containing functional groups (Figure 4a) and the charged surfaces of both biochar and Mt in interacting with the cationic BER molecule.

#### 3.3.3 Adsorption thermodynamics

The thermodynamic parameters for BER adsorption onto BC, BMC, and BMC-Mt10% at 288, 298, and 308 K are presented in Supplementary Table S4. As illustrated in Figures 5c–e, the adsorption capacity of all materials increases with temperature, indicating that higher temperatures favor BER uptake. For all materials, ΔG° values are negative across the studied temperature range, indicating that BER adsorption is spontaneous. Furthermore, ΔG° values become more negative with increasing temperature, which indicates that higher temperatures enhance the driving force for adsorption, increasing spontaneity. Notably, the ΔG° values for BMC-Mt10% are substantially more negative than those for BC and BMC, reflecting its superior adsorption affinity and capacity for BER.

The positive ΔH° values for all materials confirm the endothermic nature of the adsorption process. This endothermicity explains the enhanced adsorption capacity at elevated temperatures, as increased thermal energy facilitates overcoming energy barriers and promotes diffusion of BER into internal pores. According to established criteria, ΔH° values < 20 kJ·mol^-1^ indicate physisorption, 20–80 kJ·mol^-1^ indicate physico-chemical adsorption, and >80 kJ·mol^-1^ indicate chemisorption (Luo et al., 2022). Based on ΔH° values (26.66, 94.16, and 192.9 kJ·mol^-1^ for BC, BMC, and BMC-Mt10%, respectively), BC exhibits physico-chemical adsorption, whereas BMC and BMC-Mt10% show dominant chemisorption characteristics. The high ΔH° for BMC-Mt10% indicates that chemical interactions, such as hydrogen bonding, π-π conjugation, and potential surface complexation, govern adsorption on this composite. This conclusion is consistent with the kinetic analysis (Figure 5a; Table 2), where the pseudo-second-order model suggests chemisorption as the rate-limiting step.

Positive ΔS° values for all materials indicate increased randomness at the solid–liquid interface during adsorption. In aqueous systems, adsorption sites are initially occupied by water molecules through hydration before BER approaches the surface. The system becomes more ordered as water molecules organize on the adsorbent surface. When BER molecules diffuse into pores and interact with active sites, they displace pre-adsorbed water molecules. Crucially, as water molecules are significantly smaller than the bulky BER molecules, the adsorption of a single BER molecule necessitates the desorption of multiple water molecules. This increase in free species in solution results in higher translational entropy, promoting the spontaneity of the endothermic adsorption process (Yang et al., 2016; Zhou et al., 2020). This displacement releases water molecules into the bulk solution, increasing their mobility and overall system entropy. The higher ΔS° value for BMC-Mt10% suggests a more hydrated surface, likely due to the hydrophilic nature of Mt. This leads to displacement of more water molecules per adsorbed BER molecule, enhancing the entropic driving force. The combination of positive ΔH° and ΔS° indicates that adsorption is both enthalpically and entropically driven, with entropy playing a greater role in modified materials. The thermodynamic parameters indicate that BMC-Mt10% exhibits the most favorable adsorption behavior, with the most negative ΔG°, highest ΔH°, and largest ΔS°. These findings support the enhanced affinity and adsorption performance of BMC-Mt10% toward BER, consistent with kinetic and isotherm results. The strong chemisorption character, particularly for BMC-Mt10%, further supports the proposed multi-mechanism adsorption involving hydrogen bonding, π-π interactions, and electrostatic forces.

#### 3.3.4 Regeneration adsorption performance

Regenerative adsorption performance is a key factor in determining the potential applications of the material. The desorption and regeneration results for the three materials treated with 0.5 mol·L^-1^ NaOH are presented in Figure 5f. The adsorption performance of BC and BMC decreases with increasing regeneration cycles. After five cycles, the adsorption capacity of BC decreases to 39.27% of its initial value. This decline is attributed to the reliance of BC on chemical adsorption; repeated elution leads to the loss of surface functional groups and reduced adsorption performance (Hu et al., 2021). After five desorption cycles, BMC demonstrated a 65.85% retention of its initial adsorption capacity, indicating a notable enhancement in its material performance. This improvement is attributed to increased functional groups and larger specific surface area after ball-milled. After five desorption cycles, BMC-Mt10% retains 76.99% of its initial adsorption capacity. This suggests that the composite exhibits enhanced stability and that Mt is not merely deposited on the biochar surface but is integrated in a more stable form. In summary, BMC-Mt10% exhibits excellent regeneration performance and is a stable, high-performance adsorbent.

#### 3.3.5 Effects of environmental factors

The practical application of adsorbents in real wastewater systems requires understanding how environmental variables influence adsorption performance. Two critical factors—solution pH and coexisting natural organic matter (humic acid, HA)—were investigated for their effects on BER adsorption onto BC, BMC, and BMC-Mt10%, with results presented in Supplementary Figure S1.

Solution pH influences the surface charge of the material and the ionization state of the adsorbate, playing a key role in adsorption (Luo et al., 2022; Xu et al., 2022). BER contains polar groups such as N_3_^−^ and –OH and has a dissociation constant (pKa) of 11.5, reflecting its acid–base properties and solubility. Therefore, BER predominantly exists in its cationic form (BER^+^) due to the permanent positive charge on its quaternary ammonium group. As shown in Supplementary Figure S1b, adsorption capacity initially increases and then decreases with pH. The possible reason is that, under acidic conditions, protonated BER forms hydrogen bonds with functional groups on the biochar surface (Shareef et al., 2006; Song et al., 2018). Furthermore, interactions between interlayer water in BMC-Mt10% and ionized species contribute to enhanced adsorption. In alkaline solutions, deprotonation of hydroxyl and carboxyl groups increases electrostatic repulsion between BER anions, thereby inhibiting adsorption and reducing capacity. Among the samples, BC shows the greatest decline in adsorption capacity, decreasing from 17.26 to 0.869 mg·g^-1^. In comparison, the adsorption capacity of BMC decreases from 108.3 to 95.19 mg·g^-1^, while that of BMC-Mt10% decreases from 209.3 to 184.5 mg·g^-1^.

Previous studies have shown that humic acid can modify the physicochemical properties of carbon materials, enhancing adsorption of organic pollutants. As shown in Supplementary Figure S1b, the adsorption capacity of BC and BMC decreases with increasing humic acid concentration This reduction is attributed to humic acid, as a bulky macromolecule, competing for and occupying surface active sites. This blocks pores and reduces effective surface area, thereby limiting BER accessibility (Liu et al., 2021). Furthermore, humic acid interacts with surface functional groups, further reducing adsorption capacity. Interestingly, at low humic acid concentrations (≤10 mg), BMC-Mt10% shows a slight increase in BER adsorption capacity. This effect may be attributed to the formation of an “HA bridge” on the composite surface, where small amounts of HA introduce additional functional groups that serve as anchoring sites for BER via electrostatic interactions or hydrogen bonding. This phenomenon may result from abundant polar groups, such as -OH, present on BMC-Mt10%, which facilitate HA adsorption via hydrogen bonding or electrostatic interactions. The adsorbed HA can then interact with BER through hydrogen bonding, creating additional adsorption sites (Kim et al., 2020).

### 3.4 Analysis of adsorption mechanism

#### 3.4.1 Raman spectroscopy analysis

**FIGURE 6:**
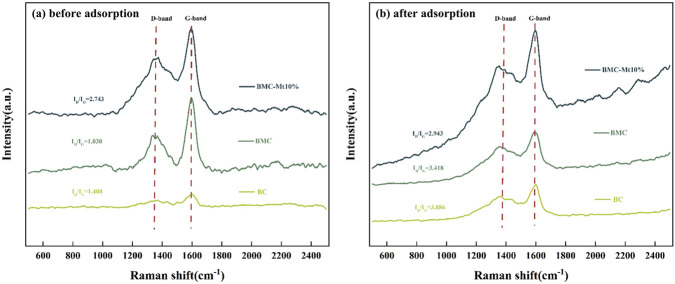
Raman spectra of BC, BMC, and BMC-Mt10% **(a)** before and **(b)** after berberine adsorption.

Raman spectroscopy is used to probe the carbon structure of materials and detect changes in electronic configuration upon adsorbate interaction. Figure 6 illustrates the Raman spectra of three adsorbents, showing vibrational characteristics before and after adsorption. Distinct D and G bands are observed in all samples. The presence of these bands indicates a disordered carbon microstructure, while their overlap suggests a significant amount of amorphous carbon, consistent with XRD results (Figure 4b). The intensity ratio (ID/IG) is inversely correlated with the degree of graphitization and structural order (Inam et al., 2020). Before adsorption, the ID/IG ratio follows: BMC-Mt10% > BC > BMC > 1. This indicates that all materials exhibit some degree of graphitization. The lower ID/IG ratio of BMC compared to BC suggests that ball milling alone may increase the ordering of certain carbon, possibly through the removal of amorphous components or the enhancement of aromatic functional groups. Notably, BMC-Mt10% exhibits the highest ID/IG ratio among the three materials, which can be attributed to the synergistic effects of Mt incorporation and the co-ball milling process. The introduction and fragmentation of Mt during ball milling disrupt the ordered carbon framework, generating structural defects, edge sites, and a complex porous structure. These defects provide additional active sites, contributing to enhanced adsorption by offering more binding sites for BER. Comparison of Figures 6a,b shows that the ID/IG ratios of all materials increase after BER adsorption. This increase is particularly pronounced for BMC-Mt10%. The increase in ID/IG ratio can be attributed to electron transfer during interaction between BER and the adsorbent surface. The surfaces of these materials are rich in oxygen-containing functional groups (-OH, -COOH) and aromatic C=C structures, which can act as strong π-electron donors or acceptors during adsorption (Gao et al., 2022). The benzene rings of BER, which are electron-rich aromatic systems, participate in π-π interactions with the graphitic domains of the adsorbent. The G band corresponds to the stretching vibration of sp^2^ carbon atoms, characteristic of aromatic structures (Wang R. et al., 2019). These interactions involve electron transfer between BER and the adsorbent, perturbing the electronic environment of sp^2^ carbon atoms and increasing the ID/IG ratio (Zhang et al., 2011). This spectroscopic evidence supports the involvement of π-π electron donor-acceptor interactions as a key mechanism in BER adsorption.

#### 3.4.2 X-ray photoelectron spectroscopy (XPS) analysis

**FIGURE 7:**
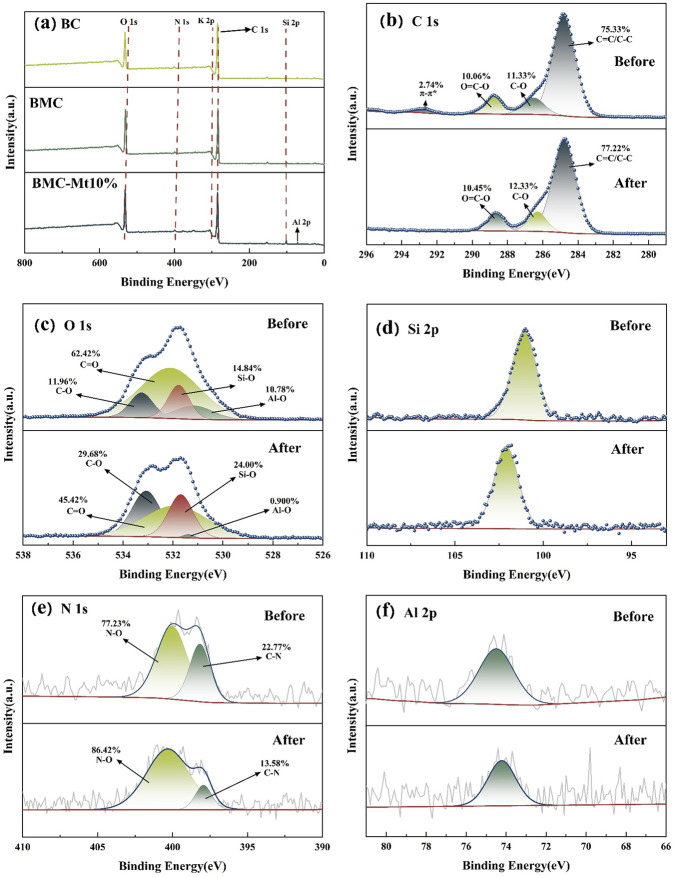
XPS spectra of BMC-Mt10% before and after berberine (BER) adsorption: **(a)** survey spectra; high-resolution spectra of **(b)** C 1s, **(c)** O 1s, **(d)** Si 2p, **(e)** N 1s, and **(f)** Al 2p.

XPS analysis was conducted to investigate changes in surface elemental composition and chemical states before and after BER adsorption. Figure 7a shows the survey spectra, while Figures 7b–f shows high-resolution spectra of C 1s, O 1s, Si 2p, Al 2p, and N 1s. The survey spectra confirm successful formation of the composite (Figure 7a). Compared with BC and BMC, BMC-Mt10% exhibits distinct Al 1s and enhanced Si 2p peaks, confirming the integration of Mt into the biochar matrix.

The high-resolution C 1s spectrum of BMC-Mt10% before adsorption can be deconvoluted into four components: π–π* satellite, O=C-O, C-O, and C=C/C-C, corresponding to different carbon functional groups (Figure 7b). Notably, the π–π satellite peak at 292.7 eV disappears after BER adsorption. Meanwhile, the relative proportions of O=C-O, C-O, and C=C/C-C increase. These spectral changes indicate π-π interactions between BER and the composite surface. Aromatic structures on BMC-Mt10% can act as π-electron acceptors, while BER acts as a π-electron donor (Kim and Hyun, 2018). The electron transfer from BER to the adsorbent surface during π-π conjugation disrupts the shake-up satellite feature, leading to its disappearance. Simultaneously, electron redistribution also alters the binding energies of other carbon species, resulting in increased proportions of the corresponding peaks. These results are consistent with Raman data (Figure 6), where the ID/IG ratio increases due to perturbation of the sp^2^ carbon network.

As shown in Figure 7c, the O 1s peak can be deconvoluted into C-O, C=O, Si-O, and Al-O. During adsorption, the proportion of C=O decreases, likely due to bond cleavage and transformation into C-O and other functional groups (Smith et al., 2016). The proportion of Al-O decreases significantly, possibly due to structural changes in Mt during ball milling, which may generate oxygen vacancies or surface hydroxyl groups. The nitrogen bases in BER, typically exhibiting electrophilic properties, facilitate its adsorption onto the surface, consequently contributing to the reduction in the Al-O ratio. As shown in Figure 7f, the intensity of the Al 2p peak decreases significantly after adsorption, indicating active involvement of Al in the process. The Si 2p and Al 2p spectra provide further evidence for the participation of Mt. The Si 2p spectrum (Figure 7d) shows a shift in binding energy after BER adsorption, and the spectral intensity distribution changes. This shift indicates a change in the chemical environment of silicon atoms, likely due to interactions between Si-O groups and BER molecules. Specifically, the oxygen-containing functional groups of BER may form hydrogen bonds or coordination interactions with Si-O group on the composite surface, leading to the observed binding energy shift. The Al 2p spectrum (Figure 7f) shows a marked decrease in peak intensity after adsorption, indicating the active role of aluminum. This reduction indicates that aluminum sites are involved in binding BER, consistent with the decreased Al-O proportion in the O 1s spectrum. The involvement of Al^3+^ sites suggest that ion exchange may be an important mechanism, in which BER^+^ exchanges with interlayer cations in Mt.

The N 1s spectrum of BMC-Mt10% after adsorption (Figure 7e) can be deconvoluted into two peaks assigned to N-O and C-N. The appearance and variation of these nitrogen peaks confirm the adsorption of BER, which contains nitrogen. Changes in peak area before and after adsorption reflect site occupation by BER and interactions between its nitrogen-containing groups and the adsorbent surface.

The following ion exchange reactions (Equations 10–14) are proposed during adsorption:

$${Mt-Al}^{3+}+{3NH}^{2+}⇌Mt-{NH}^{4+}+{Al\left(OH\right)}_{3}$$ (10)

$$Mt-{Na}^{+}+{NH}^{2+}⇌Mt-{NH}^{2+}+{Na}^{+}$$ (11)

$${Mt-Mg}^{2+}+{2NH}^{2+}⇌Mt-2NH2+{Mt-2NH}^{2+}+{Mg}^{2+}$$ (12)

$${Mt-Na}^{+}+H^{+}⇌{Mt-H}^{+}+{Na}^{+}$$ (13)

$${Mt-Mg}^{2+}+{2H}^{+}⇌{Mt-2H}^{+}+{Mg}^{2+}$$ (14)

While Na^+^ and Mg^2+^ may also participate in exchange reactions, their lower content in the composite compared to Al^3+^ makes aluminum-mediated exchange a dominant contributor to the overall adsorption process.

#### 3.4.3 Multifaceted adsorption mechanism

**FIGURE 8:**
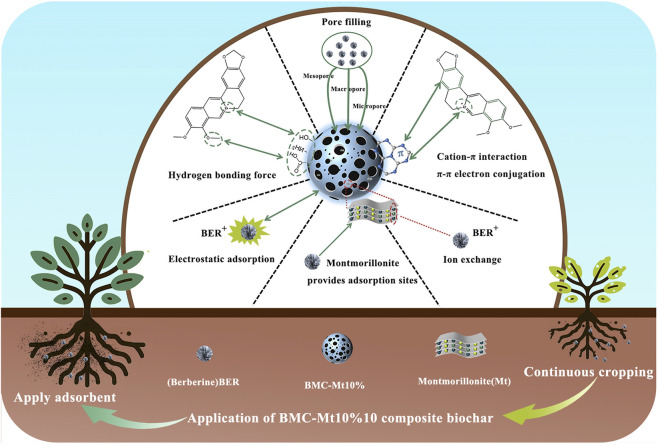
Schematic illustration of the proposed synergistic adsorption mechanism of berberine onto BMC-Mt10%.

Based on the systematic characterization and adsorption behavior studies presented above, the superior adsorption performance of BMC-Mt10% toward BER can be attributed to the synergistic interplay of multiple mechanisms, which are schematically illustrated in Figure 8. First, the hierarchical porous architecture generated by the co-ball milling process (specific surface area of 12.71 m^2^·g^-1^, a 9.1-fold increase over pristine biochar) provides ample space for BER accommodation and rapid diffusion channels, thereby establishing the structural foundation for pore filling. Spectroscopic evidence (FT-IR, XPS) demonstrates that the abundant oxygen-containing functional groups (-OH, -COOH) on the composite surface engage in strong hydrogen bonding force with BER molecules, contributing significantly to BER capture. The increase in the ID/IG ratio observed in Raman spectra after adsorption, coupled with the disappearance of the π-π* satellite peak in the C 1s XPS spectrum, provides unequivocal evidence for π-π electron conjugation between the aromatic structures. pH-dependent experiments reveal the significant contribution of electrostatic adsorption to the adsorption process, while the marked decrease in Al 2p peak intensity and Al-O proportion after adsorption, together with the shift in Si 2p binding energy, confirms the involvement of ion exchange (particularly involving Al^3+^) and surface complexation mechanisms. In summary, BER adsorption onto BMC-Mt10% proceeds through a multi-step synergistic process: BER molecules first rapidly access the hierarchical pore network via pore filling, followed by firm capture through multiple chemical interactions including hydrogen bonding force, π-π electron conjugation, electrostatic adsorption, ion exchange, and surface complexation. Consequently, co-ball milling represents a promising modification strategy that significantly enhances the BER adsorption capacity of biochar-montmorillonite composites by constructing a multifunctional platform integrating physical confinement with multiple chemical binding sites.

#### 3.4.4 Cost-effectiveness analysis of BMC-Mt10%

To evaluate the feasibility of BMC-Mt10% for large-scale soil or wastewater treatment, a comprehensive cost analysis was conducted (Supplementary Table S5). Labor costs were neglected in this estimation. Since the traditional Chinese medicine residue is an agricultural waste by-product, its raw material cost is considered negligible. Therefore, the main production costs of BMC-Mt10% include montmorillonite (Mt) raw material (0.73 USD/kg) and energy consumption during the pyrolysis process (4.014 USD/kg). For comparison, the current market prices of commercially available adsorbents are 46.26 USD/kg for activated carbon and 70.46 USD/kg for carbon nanotube materials (Shanghai McLin Biochemical Technology Co., Ltd.). The total estimated production cost of 1 kg of BMC-Mt10% is therefore approximately 3.479 USD, indicating that BMC-Mt10% is a high-performance, low-cost adsorbent suitable for large-scale application.

#### 3.4.5 Limitations and future perspectives

Despite the promising performance and cost-effectiveness of BMC-Mt10%, several limitations remain to be addressed in future research. First, while the co-ball milling strategy is theoretically applicable to alternative agricultural wastes (e.g., rice husk, corn straw), the inherent heterogeneity of biomass—driven by species and seasonal variations—poses challenges for maintaining consistent chemical composition and porosity in industrial-scale production. Future studies should focus on optimizing pyrolysis parameters for diverse feedstocks to ensure product stability. Furthermore, while chemical regeneration effectively extends the operational lifespan of the sorbent, the management of the resulting concentrated secondary liquid waste necessitates the development of more refined terminal treatment protocols to eliminate potential ecological hazards. Subsequent research should prioritize pilot-scale experiments to rigorously evaluate the long-term environmental behavior, stability, and ecological fate of these composites within complex soil ecosystems, thereby providing robust data support for their large-scale engineering application in remediating continuous cropping obstacles.

## 4 Conclusion

In this study, a high-performance biochar–montmorillonite composite (BMC-Mt10%) was successfully engineered via a one-step co-ball milling strategy using traditional Chinese medicine residue as a precursor for the efficient removal of BER. The findings demonstrate that co-ball milling synergistically enhances the physicochemical properties of the composite, resulting in a 9.1-fold increase in specific surface area compared to pristine biochar, which is attributed to the effective exfoliation of Mt lamellae and the structural fragmentation of the biochar matrix. The composite exhibited an exceptional maximum adsorption capacity of 231.60 mg·g^-1^, significantly outperforming its individual constituents. Adsorption kinetic studies revealed a rapid process following the pseudo-second-order model, while thermodynamic analyses confirmed the reaction to be spontaneous and endothermic. Detailed characterization and mechanistic investigations indicated that the superior performance of BMC-Mt10% arises from the synergistic integration of multiple interactions, including pore filling, electrostatic attraction, cation exchange, π-π stacking, and hydrogen bonding. Furthermore, the material displayed robust structural stability, maintaining 76.99% of its initial adsorption capacity after five consecutive regeneration cycles. Overall, this research provides a sustainable “waste-treat-waste” paradigm, demonstrating that BMC-Mt composites offer a cost-effective and environmentally friendly solution for mitigating allelochemical-induced continuous cropping obstacles, thereby contributing to the high-value valorization of traditional Chinese medicine residue waste in modern agricultural applications.

## Data Availability

The original contributions presented in the study are included in the article/Supplementary Material, further inquiries can be directed to the corresponding author.

## References

[B1] Ali W. A. Richards S. E. Alzard R. H. (2025). Unlocking the potential of ball milling for nanomaterial synthesis: an overview. J. Industrial Eng. Chem. 149, 63–93. 10.1016/j.jiec.2025.01.054

[B2] Benhouria A. Islam M. A. Zaghouane-Boudiaf H. Boutahala M. Hameed B. H. (2015). Calcium alginate–bentonite–activated carbon composite beads as highly effective adsorbent for methylene blue. Chem. Eng. J. 270, 621–630. 10.1016/j.cej.2015.02.030

[B3] Cao D.-Q. Yang W.-Y. Wang Z. Hao X.-D. (2019). Role of extracellular polymeric substance in adsorption of quinolone antibiotics by microbial cells in excess sludge. Chem. Eng. J. 370, 684–694. 10.1016/j.cej.2019.03.230

[B4] Cao P. Wu S. Wu T. Deng Y. Zhang Q. Wang K. (2020). The important role of polysaccharides from a traditional Chinese medicine-lung cleansing and detoxifying decoction against the COVID-19 pandemic. Carbohydr. Polym. 240, 116346. 10.1016/j.carbpol.2020.116346 32475597 PMC7175912

[B5] Chen B. Zhou D. Zhu L. (2008). Transitional adsorption and partition of nonpolar and polar aromatic contaminants by biochars of pine needles with different pyrolytic temperatures. Environ. Sci. and Technol. 42, 5137–5143. 10.1021/es8002684 18754360

[B6] Chen L. Chen X. L. Zhou C. H. Yang H. M. Ji S. F. Tong D. S. (2017). Environmental-friendly montmorillonite-biochar composites: facile production and tunable adsorption-release of ammonium and phosphate. J. Clean. Prod. 156, 648–659. 10.1016/j.jclepro.2017.04.050

[B7] Chen J. Yu Q. Li M. Sun S. Zhan D. Wang Y. (2023). Preparation of high specific surface area activated carbon fiber by high-temperature vacuum activation and its superior water vapor adsorption for air humidity control. J. Mater. Sci. 58, 2469–2493. 10.1007/s10853-023-08205-z

[B8] Cho S.-H. Lee S. Kim Y. Song H. Lee J. Tsang Y. F. (2023). Applications of agricultural residue biochars to removal of toxic gases emitted from chemical plants: a review. Sci. Total Environ. 868, 161655. 10.1016/j.scitotenv.2023.161655 36649775

[B9] Dai H. Liu Z. Yu J. Teng X. Liu L. Jia M. (2025a). Assessment of the characters of a novel phosphoric acid and mineral-comodified biochar composite and its potential application in saline–alkali soil improvement. Agriculture 15, 785. 10.3390/agriculture15070785

[B10] Dai H. Jia M. Xue J. Huang Y. Yu J. (2025b). Effects of different modified biochars on growth of Kosteletzkya virginica and corresponding transcriptome analysis. Plants 14, 1849. 10.3390/plants14121849 40573837 PMC12196827

[B11] Ejder-Korucu M. Gürses A. Karaca S. (2016). Poly(ethylene oxide)/clay nanaocomposites: thermal and mechanical properties. Appl. Surf. Sci. 378, 1–7. 10.1016/j.apsusc.2016.03.159

[B12] El-Naggar A. Lee S. S. Rinklebe J. Farooq M. Song H. Sarmah A. K. (2019). Biochar application to low fertility soils: a review of current status, and future prospects. Geoderma 337, 536–554. 10.1016/j.geoderma.2018.09.034

[B13] Fang J. Pan J. Zhou J. Li Q. Hong B. Lv D. (2025). Crop rotation alleviates continuous cropping obstacles of lily and improves secondary metabolites in bulb through shifting rhizospheric microbiota. Sci. Hortic. 343, 114074. 10.1016/j.scienta.2025.114074

[B14] Feng Q. Wang B. Chen M. Wu P. Lee X. Xing Y. (2021). Invasive plants as potential sustainable feedstocks for biochar production and multiple applications: a review. Resour. Conservation Recycl. 164, 105204. 10.1016/j.resconrec.2020.105204

[B15] Fseha Y. H. Sizirici B. Yildiz I. (2021). The potential of date palm waste biochar for single and simultaneous removal of ammonium and phosphate from aqueous solutions. J. Environ. Chem. Eng. 9, 106598. 10.1016/j.jece.2021.106598

[B16] Gao W. Lin Z. Chen H. Yan S. Zhu H. Zhang H. (2022). Roles of graphitization degree and surface functional groups of N-doped activated biochar for phenol adsorption. J. Anal. Appl. Pyrolysis 167, 105700. 10.1016/j.jaap.2022.105700

[B17] Hou X. Deng Y. Dai M. Jiang X. Li S. Fu H. (2022). Migration and transformation of heavy metals in Chinese medicine residues during the process of traditional pyrolysis and solar pyrolysis. Chemosphere 293, 133658. 10.1016/j.chemosphere.2022.133658 35051513

[B18] Hu Z.-T. Ding Y. Shao Y. Cai L. Jin Z.-Y. Liu Z. (2021). Banana peel biochar with nanoflake-assembled structure for cross contamination treatment in water: interaction behaviors between lead and tetracycline. Chem. Eng. J. 420, 129807. 10.1016/j.cej.2021.129807

[B19] Inam A. Brydson R. Edmonds D. V. (2020). Raman spectroscopy study of the crystallinity of graphite formed in an experimental free-machining steel. Mater. Charact. 163, 110264. 10.1016/j.matchar.2020.110264

[B20] Jing F. Guan J. Tang W. Chen J. (2022). Mechanistic insight into adsorptive removal of ionic NOR and nonionic DEP organic contaminates by clay-biochar composites. Environ. Pollut. 310, 119881. 10.1016/j.envpol.2022.119881 35952988

[B21] Jung K.-W. Hwang M.-J. Jeong T.-U. Ahn K.-H. (2015). A novel approach for preparation of modified-biochar derived from marine macroalgae: dual purpose electro-modification for improvement of surface area and metal impregnation. Bioresour. Technol. 191, 342–345. 10.1016/j.biortech.2015.05.052 26008889

[B22] Kalak T. Kaczmarek M. Nowicki P. Pietrzak R. Tachibana Y. Cierpiszewski R. (2022). Preparation of nitrogen-enriched pine sawdust-based activated carbons and their application for copper removal from the aquatic environment. Wood Sci. Technol. 56, 1721–1742. 10.1007/s00226-022-01423-9

[B23] Kim J. Hyun S. (2018). Sorption of ionic and nonionic organic solutes onto giant Miscanthus-derived biochar from methanol-water mixtures. Sci. Total Environ. 615, 805–813. 10.1016/j.scitotenv.2017.09.296 28992505

[B24] Kim J. E. Bhatia S. K. Song H. J. Yoo E. Jeon H. J. Yoon J.-Y. (2020). Adsorptive removal of tetracycline from aqueous solution by maple leaf-derived biochar. Bioresour. Technol. 306, 123092. 10.1016/j.biortech.2020.123092 32163869

[B25] Kumar M. Xiong X. Wan Z. Sun Y. Tsang D. C. W. Gupta J. (2020). Ball milling as a mechanochemical technology for fabrication of novel biochar nanomaterials. Bioresour. Technol. 312, 123613. 10.1016/j.biortech.2020.123613 32513509

[B26] Li Y. Fu J. Deng S. Lu X. (2014). Optimization of mesoporous carbons for efficient adsorption of berberine hydrochloride from aqueous solutions. J. Colloid Interface Sci. 424, 104–112. 10.1016/j.jcis.2014.03.012 24767505

[B27] Li H. Kong J. Zhang H. Gao J. Fang Y. Shi J. (2023). Mechanisms and adsorption capacities of ball milled biomass fly ash/biochar composites for the adsorption of methylene blue dye from aqueous solution. J. Water Process Eng. 53, 103713. 10.1016/j.jwpe.2023.103713

[B28] Lin D. Ji J. Long Z. Yang K. Wu F. (2012). The influence of dissolved and surface-bound humic acid on the toxicity of TiO2 nanoparticles to chlorella sp. Water Res. 46, 4477–4487. 10.1016/j.watres.2012.05.035 22704133

[B29] Lin C.-C. Liu Y.-T. Chang P.-H. Hsieh Y.-C. Tzou Y.-M. (2023). Inhibition of continuous cropping obstacle of celery by chemically modified biochar: an efficient approach to decrease bioavailability of phenolic allelochemicals. J. Environ. Manag. 348, 119316. 10.1016/j.jenvman.2023.119316 37862893

[B30] Liou T.-H. Wu S.-J. (2009). Characteristics of microporous/mesoporous carbons prepared from rice husk under base- and acid-treated conditions. J. Hazard. Mater. 171, 693–703. 10.1016/j.jhazmat.2009.06.056 19595505

[B31] Liu Q. Li D. Cheng H. Cheng J. Du K. Hu Y. (2021). High mesoporosity phosphorus-containing biochar fabricated from Camellia oleifera shells: impressive tetracycline adsorption performance and promotion of pyrophosphate-like surface functional groups (C-O-P bond). Bioresour. Technol. 329, 124922. 10.1016/j.biortech.2021.124922 33713899

[B32] Liu X. Shi S. Wang S. Ren Z. Wang Y. Guo Q. (2025). Ball-milled biochar-vermiculite/zeolite magnetic composites for adsorption of lead and p-nitrophenol from wastewater: synthesis, performance, and mechanisms. Environ. Res. 286, 122761. 10.1016/j.envres.2025.122761 40912630

[B33] Lu Q. Li C. (2021). Comprehensive utilization of Chinese medicine residues for industry and environment protection: turning waste into treasure. J. Clean. Prod. 279, 123856. 10.1016/j.jclepro.2020.123856

[B34] Luo L. Yang C. Yang X. Liu F. Wang X. Chen P. (2022). Construction of ultra-microporous activated carbons derived from waste distiller's grains for efficient CO2 adsorption. Sep. Purif. Technol. 302, 122134. 10.1016/j.seppur.2022.122134

[B35] Lyu H. Gao B. He F. Zimmerman A. R. Ding C. Huang H. (2018). Effects of ball milling on the physicochemical and sorptive properties of biochar: experimental observations and governing mechanisms. Environ. Pollut. 233, 54–63. 10.1016/j.envpol.2017.10.037 29053998

[B36] Ma Y. Yang L. Wu L. Li P. Qi X. He L. (2020). Carbon nanotube supported sludge biochar as an efficient adsorbent for low concentrations of sulfamethoxazole removal. Sci. Total Environ. 718, 137299. 10.1016/j.scitotenv.2020.137299 32088478

[B37] Mariana M. Mistar E. M. Alfatah T. Supardan M. D. (2021). High-porous activated carbon derived from Myristica fragrans shell using one-step KOH activation for methylene blue adsorption. Bioresour. Technol. Rep. 16, 100845. 10.1016/j.biteb.2021.100845

[B38] Moore V. M. Schlautman B. Fei S. Roberts L. M. Wolfe M. Ryan M. R. (2022). Plant breeding for intercropping in temperate field crop systems: a review. Front. Plant Sci. 13, 843065. 10.3389/fpls.2022.843065 35432391 PMC9009171

[B39] Nolte M. W. Shanks B. H. (2017). A perspective on catalytic strategies for deoxygenation in biomass pyrolysis. Energy Technol. 5, 7–18. 10.1002/ente.201600096

[B40] Petrovic B. Gorbounov M. Masoudi Soltani S. (2022). Impact of surface functional groups and their introduction methods on the mechanisms of CO2 adsorption on porous carbonaceous adsorbents. Carbon Capture Sci. and Technol. 3, 100045. 10.1016/j.ccst.2022.100045

[B41] Sathasivam J. Rajaraman P. V. Narayanasamy S. (2025). Assessment of cerium adsorption potential of phosphoric acid activated biochar in aqueous system: modelling and mechanistic insights. Environ. Res. 264, 120301. 10.1016/j.envres.2024.120301 39505131

[B42] Shareef A. Angove M. J. Wells J. D. Johnson B. B. (2006). Sorption of bisphenol A, 17α-ethynylestradiol and estrone to mineral surfaces. J. Colloid Interface Sci. 297, 62–69. 10.1016/j.jcis.2005.10.039 16298385

[B43] Shu Q. Gao J. Nawaz Z. Liao Y. Wang D. Wang J. (2010). Synthesis of biodiesel from waste vegetable oil with large amounts of free fatty acids using a carbon-based solid acid catalyst. Appl. Energy 87, 2589–2596. 10.1016/j.apenergy.2010.03.024

[B44] Smith M. Scudiero L. Espinal J. McEwen J.-S. Garcia-Perez M. (2016). Improving the deconvolution and interpretation of XPS spectra from chars by *ab initio* calculations. Carbon 110, 155–171. 10.1016/j.carbon.2016.09.012

[B45] Song J. Y. Bhadra B. N. Khan N. A. Jhung S. H. (2018). Adsorptive removal of artificial sweeteners from water using porous carbons derived from metal azolate framework-6. Microporous Mesoporous Mater. 260, 1–8. 10.1016/j.micromeso.2017.10.021

[B46] Song Y. Guo H. Qiu Z. Lin C. Zhao X. Geng X. (2025). Study on phosphorus adsorption properties of mechanical ball milling modified biochar-zeolite composites. Desalination Water Treat. 323, 101270. 10.1016/j.dwt.2025.101270

[B47] Tang J. Zhao B. Lyu H. Li D. (2021). Development of a novel pyrite/biochar composite (BM-FeS2@BC) by ball milling for aqueous Cr(VI) removal and its mechanisms. J. Hazard. Mater. 413, 125415. 10.1016/j.jhazmat.2021.125415 33626470

[B48] Vakili A. Zinatizadeh A. A. Rahimi Z. Zinadini S. Mohammadi P. Azizi S. (2023). The impact of activation temperature and time on the characteristics and performance of agricultural waste-based activated carbons for removing dye and residual COD from wastewater. J. Clean. Prod. 382, 134899. 10.1016/j.jclepro.2022.134899

[B49] Wang J. Xu C. Wong Y. K. Li Y. Liao F. Jiang T. (2019). Artemisinin, the magic drug discovered from traditional Chinese medicine. Engineering 5, 32–39. 10.1016/j.eng.2018.11.011

[B50] Wang R. An H. Zhang H. Zhang X. Feng J. Wei T. (2019). High active radicals induced from peroxymonosulfate by mixed crystal types of CuFeO2 as catalysts in the water. Appl. Surf. Sci. 484, 1118–1127. 10.1016/j.apsusc.2019.04.182

[B51] Wang J. Tian T. Wang H. Cui J. Shi X. Song J. (2022). Chitosan-coated compound fertilizer application and crop rotation alleviate continuous cotton cropping obstacles by modulating root exudates. Rhizosphere 23, 100581. 10.1016/j.rhisph.2022.100581

[B52] Wang F. Zhang X. Wei M. Wang Y. Liang Z. Xia P. (2022). Appropriate crop rotation alleviates continuous cropping barriers by changing rhizosphere microorganisms in Panax notoginseng. Rhizosphere 23, 100568. 10.1016/j.rhisph.2022.100568

[B53] Wu F.-C. Tseng R.-L. Juang R.-S. (2009). Characteristics of elovich equation used for the analysis of adsorption kinetics in dye-chitosan systems. Chem. Eng. J. 150, 366–373. 10.1016/j.cej.2009.01.014

[B54] Wu X. Wang J. Amanze C. Yu R. Li J. Wu X. (2022). Exploring the dynamic of microbial community and metabolic function in food waste composting amended with traditional Chinese medicine residues. J. Environ. Manag. 319, 115765. 10.1016/j.jenvman.2022.115765 35982566

[B55] Wu X. Dai D. Li N. Zheng H. Wang C. Lin W. (2025). Recycling traditional Chinese medicine residues: a review. Environ. Chem. Lett. 23, 977–997. 10.1007/s10311-025-01837-4

[B56] Xing X. Zhang Y. Zhou G. Zhang Y. Yue J. Wang X. (2023). Mechanisms of polystyrene nanoplastics adsorption onto activated carbon modified by ZnCl2. Sci. Total Environ. 876, 162763. 10.1016/j.scitotenv.2023.162763 36921872

[B57] Xu Y. Khan M. A. Wang F. Xia M. Lei W. (2018). Novel multi amine-containing gemini surfactant modified montmorillonite as adsorbents for removal of phenols. Appl. Clay Sci. 162, 204–213. 10.1016/j.clay.2018.06.023

[B58] Xu J. Zhang Y. Li B. Fan S. Xu H. Guan D.-X. (2022). Improved adsorption properties of tetracycline on KOH/KMnO4 modified biochar derived from wheat straw. Chemosphere 296, 133981. 10.1016/j.chemosphere.2022.133981 35176301

[B59] Yaghmaeiyan N. Mirzaei M. Delghavi R. (2022). Montmorillonite clay: introduction and evaluation of its applications in different organic syntheses as catalyst: a review. Results Chem. 4, 100549. 10.1016/j.rechem.2022.100549

[B60] Yang X. Xu G. Yu H. Zhang Z. (2016). Preparation of ferric-activated sludge-based adsorbent from biological sludge for tetracycline removal. Bioresour. Technol. 211, 566–573. 10.1016/j.biortech.2016.03.140 27038265

[B61] Yang Y. Sun K. Han L. Jin J. Sun H. Yang Y. (2018a). Effect of minerals on the stability of biochar. Chemosphere 204, 310–317. 10.1016/j.chemosphere.2018.04.057 29665534

[B62] Yang X. Kwon E. E. Dou X. Zhang M. Kim K.-H. Tsang D. C. W. (2018b). Fabrication of spherical biochar by a two-step thermal process from waste potato peel. Sci. Total Environ. 626, 478–485. 10.1016/j.scitotenv.2018.01.052 29353788

[B63] Yang X. Luo K. Pi Z. Shen P. Zhou P. He L. (2023). Insight to the mechanism of tetracycline removal by ball-milled nanocomposite CeO2/Fe3O4/Biochar: overlooked degradation behavior. Sep. Purif. Technol. 307, 122703. 10.1016/j.seppur.2022.122703

[B64] Yao J. Kong Q. Zhu H. Long Y. Shen D. (2014). Adsorption characteristics of nitrite on Friedel’s salt under the landfill circumstance. Chem. Eng. J. 254, 479–485. 10.1016/j.cej.2014.06.007

[B65] Zhang P. Bing X. Jiao L. Xiao H. Li B. Sun H. (2022). Amelioration effects of coastal saline-alkali soil by ball-milled red phosphorus-loaded biochar. Chem. Eng. J. 431, 133904. 10.1016/j.cej.2021.133904

[B66] Zeng L. Ma J. Yang J. Yang J. Zeng X. Zhou Y. (2024). Ball milling nano-sized biochar: bibliometrics, preparation, and environmental application. Environ. Sci. Pollut. Res. 31, 52724–52739. 10.1007/s11356-024-34777-7 39190254

[B67] Zhang S. Zhang B. Dai W. Zhang X. (2011). Oxidative damage and antioxidant responses in microcystis aeruginosa exposed to the allelochemical berberine isolated from golden thread. J. Plant Physiology 168, 639–643. 10.1016/j.jplph.2010.10.005 21131096

[B68] Zhao X. Elcin E. He L. Vithanage M. Zhang X. Wang J. (2024). Using biochar for the treatment of continuous cropping obstacle of herbal remedies: a review. Appl. Soil Ecol. 193, 105127. 10.1016/j.apsoil.2023.105127

[B69] Zhao Y. Yang S. Zhou K. Wang J. Ji C. Wang Y. (2024). Adsorption and recovery of berberine hydrochloride from wastewater by a novel magnesium-modified biochar: toward resource utilization of waste dander. J. Water Process Eng. 68, 106341. 10.1016/j.jwpe.2024.106341

[B70] Zhao J. Ji C. Peng C. Wang Y. Yang S. Li Y. (2025). Interfacial interaction mechanism between Mn doped highly conjugated biochar and berberine hydrochloride. J. Colloid Interface Sci. 677, 108–119. 10.1016/j.jcis.2024.07.147 39083888

[B71] Zhou J. Ma F. Guo H. (2020). Adsorption behavior of tetracycline from aqueous solution on ferroferric oxide nanoparticles assisted powdered activated carbon. Chem. Eng. J. 384, 123290. 10.1016/j.cej.2019.123290

[B72] Zhu L. Yang H. Zhao Y. Kang K. Liu Y. He P. (2019). Biochar combined with montmorillonite amendments increase bioavailable organic nitrogen and reduce nitrogen loss during composting. Bioresour. Technol. 294, 122224. 10.1016/j.biortech.2019.122224 31610497

