# Supplementary material for: Synergistic adsorption of berberine onto ball-milled biochar-montmorillonite composites derived from traditional Chinese medicine residue: performance, mechanism, and regeneration
Source: Front Chem. 2026 Jun 4;14:1845578. doi: 10.3389/fchem.2026.1845578 (PMC13277361; doi:10.3389/fchem.2026.1845578)
Supplement: Supplementary file 1 [file DataSheet1.docx]

Supplementary Material

**Table S1** Relevant data for analytical method validation.

**Table S2** Physical and chemical properties of BC, BMC and BMC-Mt10%.

**Table S3** Fitting parameters of intraparticle diffusion model.

**Table S4** Thermodynamic parameters of BC, BMC and BMC-Mt10% adsorption berberine.

**Table S5** Production cost breakdown of 1 kg of BMC-Mt10%.

**Figure S1** Effects of (a) initial solution pH and (b) humic acid concentration on berberine adsorption onto BC, BMC, and BMC-Mt10%.

**Table S1 Relevant data for analytical method validation.**

| **Allelochemical** | **Retention time(min)** | **Linear range(mg/L)** | **Regression equation** | **(R^2^)** | **LOQ (mg/L)** | **LOD (mg/L)** | **Precision** | **Stability** | **Recovery** | |
| --- | --- | --- | --- | --- | --- | --- | --- | --- | --- | --- |
|  |  |  |  |  |  |  | **RSD (n=5, %)** | **RSD (n=8, %)** | **Range (%)** | **RSD (n=9, %)** |
| Berberine | 3.325 | 0.391~195.7 | y=29308x-399644 | 0.999 | 0.038 | 0.019 | 0.471 | 0.203 | 99.42~107.4 | 2.651 |

**Table S2 Physical and chemical properties of BC, BMC and BMC-Mt10%.**

| **Biochar** | **Physical property** | |  | **C,H,N,O contents/%** | | | | | |  | **Atomic Ratios** | | |
| --- | --- | --- | --- | --- | --- | --- | --- | --- | --- | --- | --- | --- | --- |
|  | **Yield/%** | **Ash/%** |  | **C** | **H** | **N** | **O** | **Al** | **Si** |  | **H/C** | **O/C** | **(O+N)/C** |
| BC | 38.65 | 27.52 |  | 51.50 | 1.972 | 3.77 | 42.66 | 0.070 | 0.025 |  | 0.038 | 0.83 | 0.902 |
| BMC | — | 25.42 |  | 49.45 | 1.817 | 3.63 | 45.03 | 0.043 | 0.031 |  | 0.037 | 0.911 | 0.984 |
| BMC-Mt10% | — | 35.76 |  | 44.19 | 1.864 | 3.14 | 46.29 | 2.039 | 2.479 |  | 0.042 | 1.048 | 1.119 |

**Table S3 Fitting parameters of intraparticle diffusion model.**

| **Biochar** | **The first stage** | | |  | **The second stage** | | |  | **The third stage** | | |
| --- | --- | --- | --- | --- | --- | --- | --- | --- | --- | --- | --- |
|  | **K_1_** | **C** | **R^2^** |  | **K_2_** | **C** | **R^2^** |  | **K_3_** | **C** | **R^2^** |
| BC | 1.405 | -2.388 | 0.935 |  | 0.099 | 13.07 | 0.860 |  | - | - | - |
| BMC | 7.608 | 24.04 | 0.997 |  | 1.821 | 75.55 | 0.986 |  | 0.163 | 105.7 | 0.951 |
| BMC-Mt10% | 12.03 | 53.47 | 0.972 |  | 7.257 | 95.82 | 0.872 |  | 0.173 | 191.6 | 0.908 |

**Table S4** Thermodynamic parameters of BC, BMC and BMC-Mt10% adsorption berberine.

| **Biochar** | **∆*H*_θ_** | **∆*S*_θ_** | **∆*G*_θ_** | | |
| --- | --- | --- | --- | --- | --- |
|  |  |  | **288K** | **298K** | **308K** |
| BC | 26.66 | 58.78 | -17.49 | -18.08 | -18.66 |
| BMC | 94.16 | 283.5 | -84.38 | -87.22 | -90.05 |
| BMC-Mt10% | 192.9 | 636.5 | -189.5 | -195.8 | -202.2 |

**Table S5.** Production cost breakdown of 1 kg of BMC-Mt10%.

| **Items** | **Amount/Unit** | **Unit Price, USD** | **Total Cost, USD** |
| --- | --- | --- | --- |
| TCM Residue | 1.5 kg | 0 | 0 |
| Montmorillonite | 0.1 kg | 7.3 USD/kg | 0.73 |
| Labor Cost | 0 | 0 | 0 |
| Pyrolysis Energy | 3h | 12kw/h | 3.1375 |
| Milling/Rotation | 6h | 335w/h | 0.1464 |
| Total Estimated Cost |  |  | ≈4.014 |

Note: USD represents United States Dollars. The total cost is approximated to three decimal places. All energy costs were estimated based on the rated power of the equipment (pyrolysis furnace and ball mill) and the current local industrial electricity price.


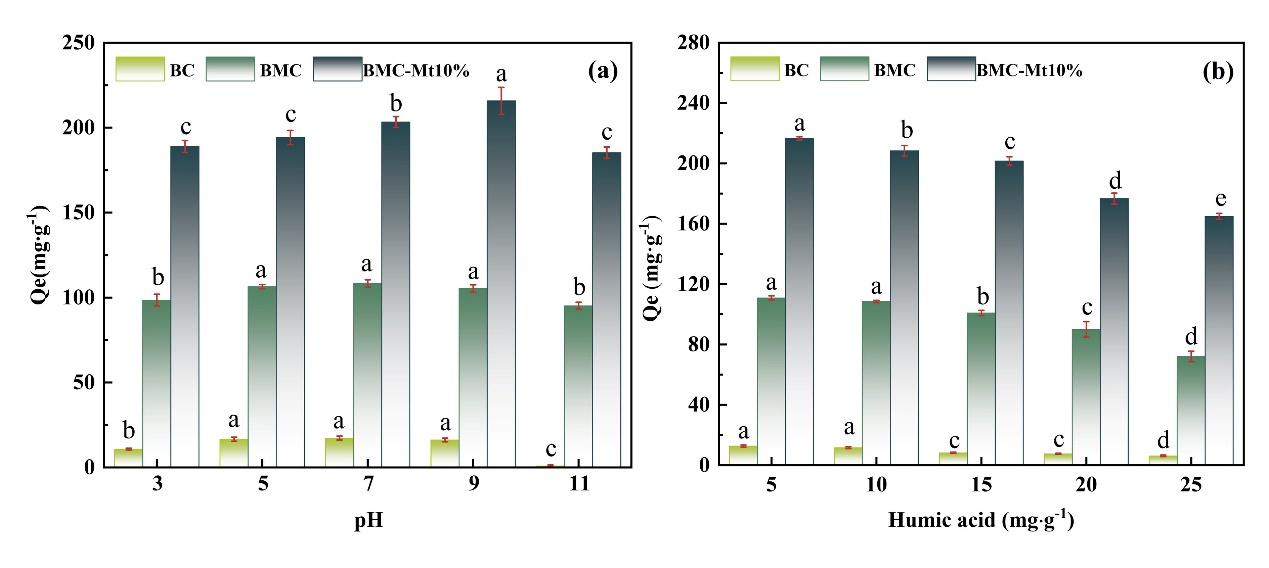


**Supplementary Figure 1.** Effects of (a) initial solution pH and (b) humic acid concentration on berberine adsorption onto BC, BMC, and BMC-Mt10%.
